# A comparative study of predicting the availability of power line communication nodes using machine learning

**DOI:** 10.1038/s41598-023-39120-7

**Published:** 2023-08-04

**Authors:** Kareem Moussa, Mennatullah Mahmoud Amin, M. Saeed Darweesh, Lobna A. Said, Abdelmoniem Elbaz, Ahmed Soltan

**Affiliations:** 1 Wireless Intelligent Networks Center (WINC), Nile University, Giza, 12677 Egypt; 2 School of Engineering and Applied Sciences, Nile University, Giza, 12677 Egypt; 3 Nanoelectronics Integrated Systems Center (NISC), Nile University, Giza, 12677 Egypt; 4 El Sewedy Electrometer Group, 6th of October City, Egypt

**Keywords:** Engineering, Electrical and electronic engineering

## Abstract

Power Line Communication technology uses power cables to transmit data. Knowing whether a node is working in advance without testing saves time and resources, leading to the proposed model. The model has been trained on three dominant features, which are SNR (Signal to Noise Ratio), RSSI (Received Signal Strength Indicator), and CINR (Carrier to Interference plus Noise Ratio). The dataset consisted of 1000 readings, with 90% in the training set and 10% in the testing set. In addition, 50% of the dataset is for class 1, which indicates whether the node readings are optimum. The model is trained with multi-layer perception, K-Nearest Neighbors, Support Vector Machine with linear and non-linear kernels, Random Forest, and adaptive boosting (ADA) algorithms to compare between statistical, vector-based, regression, decision, and predictive algorithms. ADA boost has achieved the best accuracy, F-score, precision, and recall, which are 87%, 0.86613, 0.9, 0.8646, respectively.

## Introduction

Power Line Communication (PLC) is a communication technology that uses existing power cables for data transmission. Hence, PLC is an attractive and cost-effective method for transmitting data from all devices plugged into the power plugs, such as sensors and actuators. Therefore, using PLC as a communication technology avoids adding another infrastructure for data exchange by using the power line^1–3^. Power line communication is divided into two categories based on the data rate; narrow band power line and broadband power line communications^4,5^. Narrow-band PLC is used a lot in the smart grid, by electricity companies, and in-home networks for smart home applications. Moreover, PLC is used in in-vehicle and vehicle-to-infrastructure systems, and next-generation battery management systems^6,7^. On the other hand, broadband power line communication is used in multimedia communications. Such applications are often characterized by many connected nodes, which are increasing with the Internet of Things (IoT) expansion.

The shared environment nature of the PLC raises many challenges for the communication process, such as the variable media characteristics. One issue concerns impedance matching at both the transmitter (TX) and the receiver (RX) for the PLC front end. The matching impacts the self-interference and the signal-to-self-interference-plus-noise ratio (SSINR). Typical PLC modems use a low impedance Tx path and a higher impedance Rx path in the analogue front-end for efficient harmonic distortion operation^5,7–9^. Much effort has been made for impedance matching for the PLC^10^. However, there are still challenges in the power line impedance matching due to its variable load nature.

Contemporary PLC network performance deteriorates with increasingly connected nodes. Similarly, the coexistence with neighbouring DSL networks degrades the link quality. Hence, the European Telecommunications Standards Institute (ETSI) recommends using a dynamic spectral adaptation approach^11^. Broadband PLC modems estimate the DSL-to-PLC channel interference and adapt the PLC’s transmit power spectral density accordingly. Moreover, a considerable effort has been made in PLC focused on the physical layer to deal with issues such as the time-varying behaviour of loads in electric power systems. Hence, there are dynamics and diversity of loads that result in time-frequency varying behaviour and signal attenuation when frequency and/or distance increase. Different impedance matching techniques have been illustrated in^10^. Furthermore, high power impulsive noise, impedance mismatching, the widespread use of unshielded power cables, and coupling losses impact link quality^1,4,6,11,12^. In addition, high power impulsive noises yielded by connecting and disconnecting loads, equipment, alternate current/direct current (AC/DC) converters, and electromagnetic interference due to unshielded power lines and coupling problems affect the communication media performance dynamically over time.

Research in the domain of PLC is still running to address these issues. Communities such as PRIME and G3 are developing advanced tools, techniques, methods, and approaches, such as different implementations for the MAC and PHY layers which deals with different challenges^5,9–11^. Moreover, field studies have discussed these issues^9,13^. Another technique for addressing these issues is using another communication medium, such as RF, in regions where PLC is unstable. For example, using PLC (G3-based ) with RF technology such as 6LowPAN^14^ or LoRA has been better performance^13,15,16^. However, using another technology violates the main advantage of using PLC: using existing infrastructure without added cost. Furthermore, another effort has been made to improve communication performance based on artificial intelligence (AI). So, AI is used to determine link quality and communication media quality. AI has been primarily used for RF-based technologies such as 4G/5G, optical networks, and smart cities data analysis^17–21^. Hence, this work focuses on using AI to predict link quality for a PLC-based network and determining the optimum time slot for communicating with the node via the PLC network. The used data is collected from a field configured to work with a PRIME based PLC network.

This work uses the PRIME standard to build a PLC network in the field. The nodes are implemented using PL360 PLC transceiver from Microchip technology. The network consists of 500 PLC nodes, while the data concentrator unit (DCU) is located at the transformer site. Then, a PLC sniffer located one node after the DCU point. The dataset has been collected consisting of 1000 instances of the time in which a PLC node has optimum readings of the Signal to Noise Ratio (SNR), Received Signal Strength Indicator (RSSI), and Carrier to Interference-plus-Noise (CINR). The dataset trained six models representing Statistical, Vector-based, regression, decision, and predictive algorithms. The trained statistical algorithm is adaptive boosting. The vector-based algorithms are the Support Vector Machine (SVM) linear kernel and the SVM non-linear kernel. Decision algorithms are the random forest and decision trees. Finally, the predictive algorithm is K-Nearest Neighbors.

In the rest of the paper, AI in communications is discussed in Sect. 2, and the algorithms and the dataset details are discussed in Sect. 3. Then, the behaviours of the trained models are shown in Sect. 4, with a discussion of the results in Sect. 5, and the paper is concluded in Sect. 6.

## AI in communications

AI is the field that allows computers to be smart and perform tasks that humans only did before. It has been widely developed in previous years and used in different applications. For example, AI is used for predicting some events in the future based on their historical performance, which saves time^22^.

PLC is recently being used more. It is data transmission using a Power Line Network (PLN). The problem in PLC is that PLN is not designed for this transmission, so PLC faces large noise^23,24^.

The work in^25^ has used machine learning to cluster the multi-conductor noise in PLCs to determine whether automatic clustering is helpful in this topic or not. They have used the MIMO NB noise database. They preprocessed the database to create the feature library, a Table consisting of the time segments from 5 to 500 $\mu s$ and two types of features. The first was to extract the signal, and the other was to find the relation between the two multi-conductor signal traces. The features have been evaluated to determine which are beneficial to consider. The authors have used principal component analysis (PCA) and box plots for feature evaluation. PCA reduces the dataset dimensions and keeps most of the information. A box plot displays the data on a standardized graph depending on six metrics: median, 25th percentile, 75th percentile, outlier, minimum, and maximum. The PCA shows that features 5 (Samples Skewness), 7(Samples Pearson correlation), and 9 (Distance Correlation) are the most informative features, and box-plot also shows features 5 and 7 have a visible data separation^25,26^. Three methods have been used in clustering: hierarchical clustering, self-organizing map (SOM), and clustering using representatives (CURE). Hierarchical clustering sets for each point of a cluster, calculate the distances between the clusters, combine the nearest two clusters into one cluster and then redo the process till all the clusters are combined into one cluster, forming a dendrogram which is clusters’ tree^27^. On the other hand, in CURE, a subset of the data representing C clusters is selected. For each cluster, some points far from each other are selected, then they are moved by 20% to the cluster centroid, then the algorithm merges every two clusters having two nearby representative points and then clusters all data points^28^. Finally, SOM is a network of mapped units that each unit refers to a cluster such that the larger the number of units, the more accurate the separation of data^29^. The clusters have been labeled according to the probability density functions (PDFs), which led to 35% of the data being normal, 23% being Middleton Class A, 27% being Alpha STable, 13% Generalized Extreme Value, and 2% of unknown classes. It is worth mentioning that more than five conventional noise classes were needed to represent the nature of the noise, especially in a noisy network such as a PLC environment^29^.

The noise affects the PLC node, affecting data transfer reliability. AI can be used to detect whether a node is working at a specific time or not. This can be done by knowing the readings of the node in the past and training the AI model on these readings, which leads to the prediction of the time intervals where the readings of the PLC nodes are not optimal. This prediction will lead to the early selection of other nodes for the transmission instead of testing each node to determine the functioning nodes^30^.

## Methodology

In this section, the trained machine learning algorithms, which are Multi-Layer Perception, K-Nearest Neighbour, Support Vector Machine, Random Forest, and Adaptive Boosting, are discussed along with the key information of the collected data.

### Machine learning algorithms

#### Multi-layer perception (MLP)

**Figure 1:**
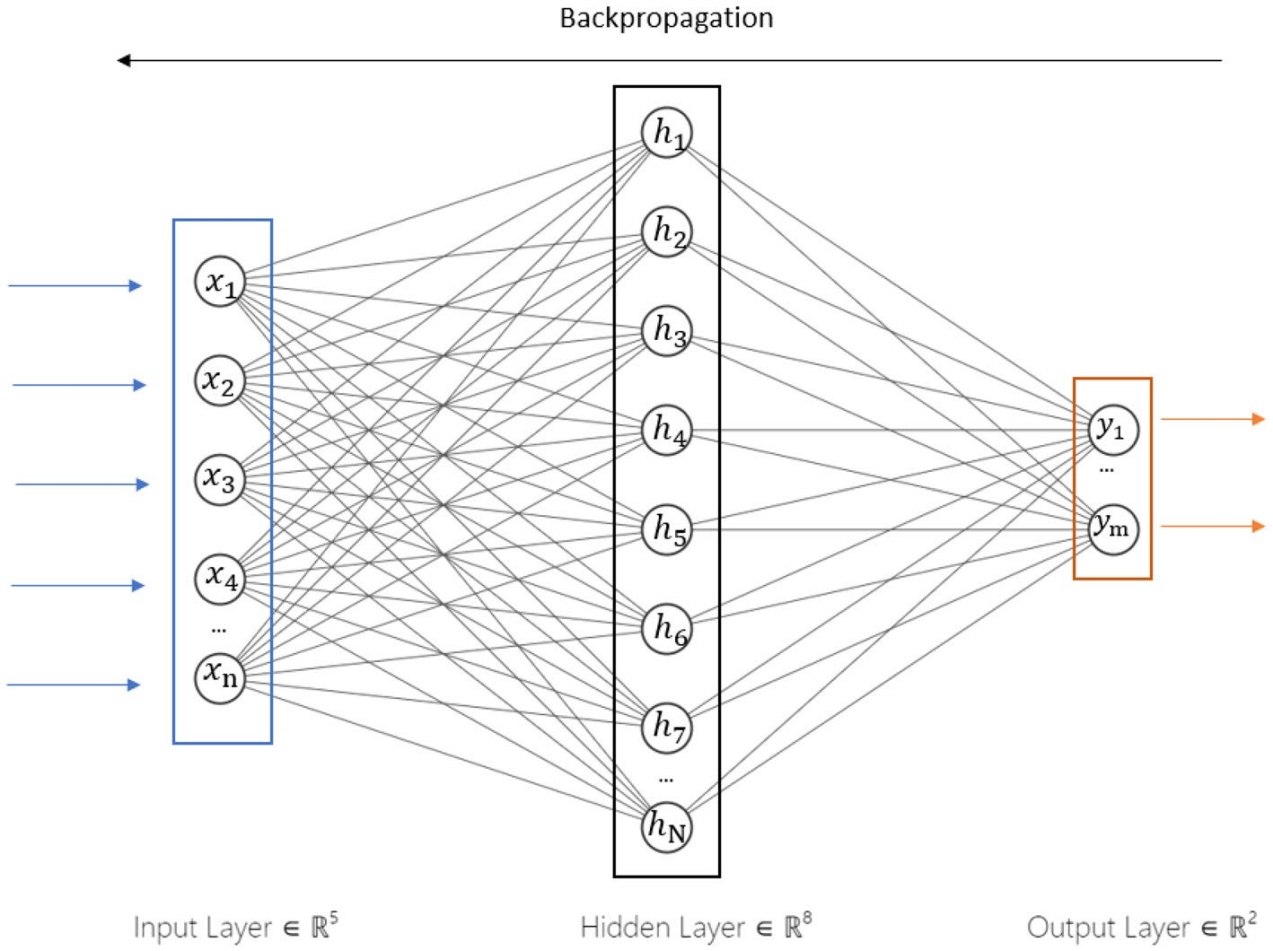
Relation between different layers of the MLP.

Multi-Layer Perception (MLP) is a neural network that is a supervised learning technique. The MLP consists of six layers: the input layer, four hidden layers, and the output layer, as depicted in Fig. 1. All the non-input nodes are neurons that use a nonlinear activation function.

#### K-nearest neighbour (KNN)

**Figure 2:**
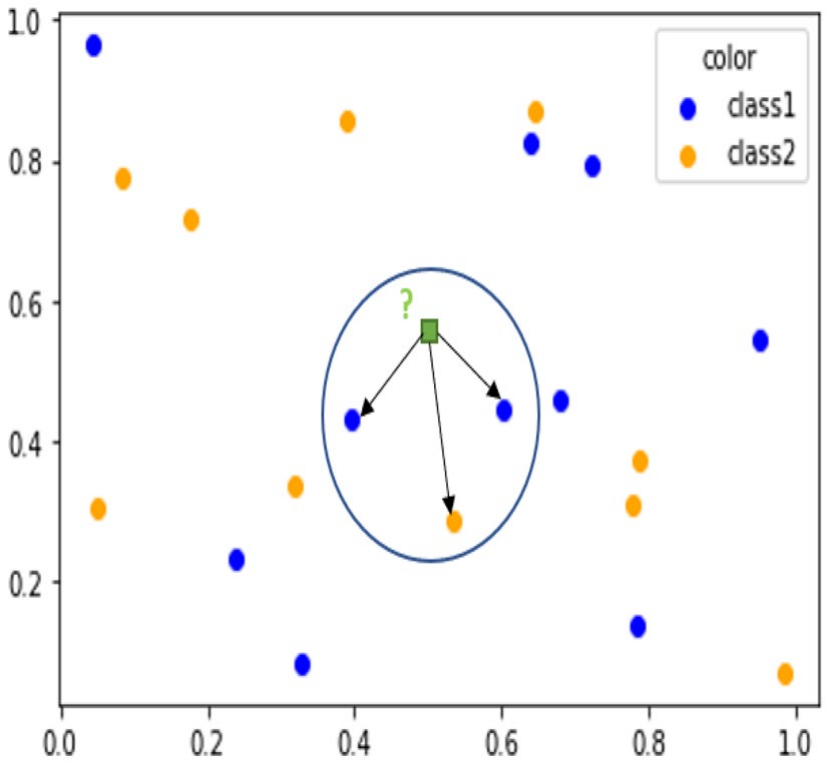
K-Nearest Neighbors, which shows the selection of the most similar k points to the input point.

K-Nearest Neighbour (KNN) is an algorithm that predicts the input class based on voting for the most similar training data instances to the input. This takes the majority class of the K nearest similar neighbours without a learning process. As shown in Fig. 2, the green circle next to the question mark is the input that is not labelled. The two red triangles and a blue square are next to the input circle because they are similar; in other words, their features are similar to the input’s features. In this example, the value of the K is chosen to be three, so the black circle contains the nearest three instances to the input. After knowing the voting participants, the majority class will be the class of the input, so the prediction of the input class is the red triangle class

where the blue square is class 1, the red triangle is class 0, and the green circle is the input.

#### Support vector machine (SVM)

**Figure 3:**
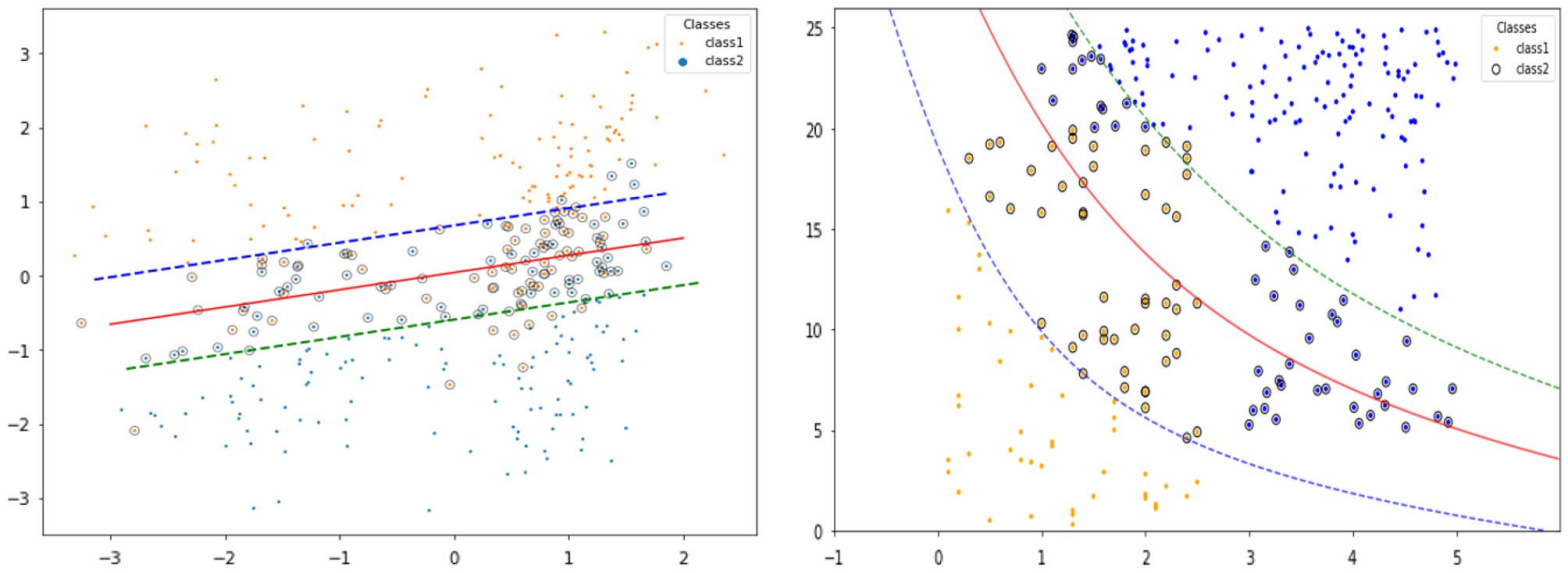
Diagram for the Support Vector Machine which shows the classification using the non-linear and linear kernels^34^.

SVM separates the data points into another readily separable dimension using a kernel. For example, as shown in Fig. 3, there are two features, x1 and x2, and two classes, black and white dots. To be able to identify which combination of the feature values would refer to the class, the feature values of each instance have been plotted, and using a non-linear kernel as part (a) and linear kernel as part (b), the parts of the plot indicating each class could be known. The hyperplane is the plane that separates the classes in n-dimensional space. The more it is farther from the data points, the more accurate the classification^31–33^.

#### Random forest

**Figure 4:**
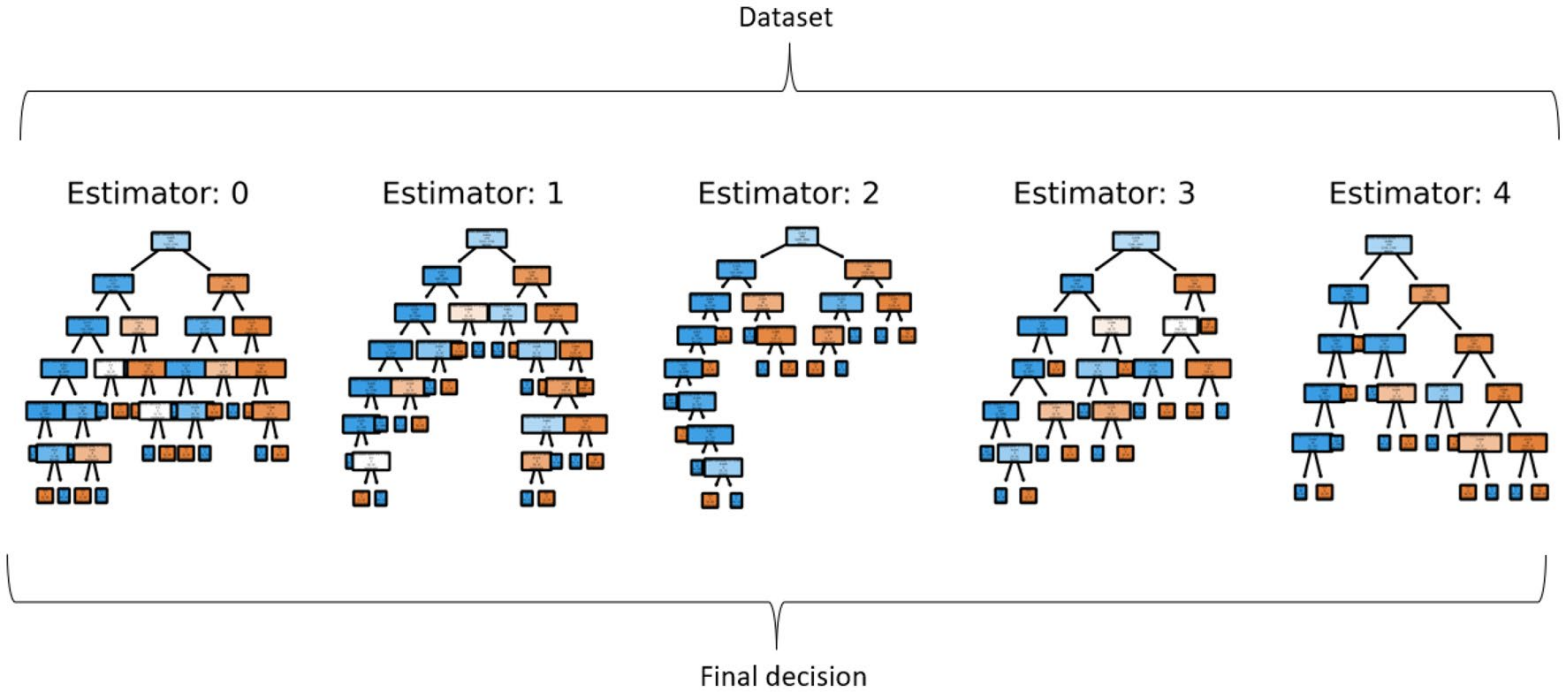
Random forest^35,37^.

The Random Forest algorithm is a group of decision trees, as shown in Fig. 4. Each of these decision trees is trained on a subset of the dataset. These portions are equally distributed. When an input is given to the random forest algorithm, each tree, based on its training, gives a classification for this input. The class with the majority of predictions is input predicted class^35,36^.

#### Adaptive boosting

**Figure 5:**
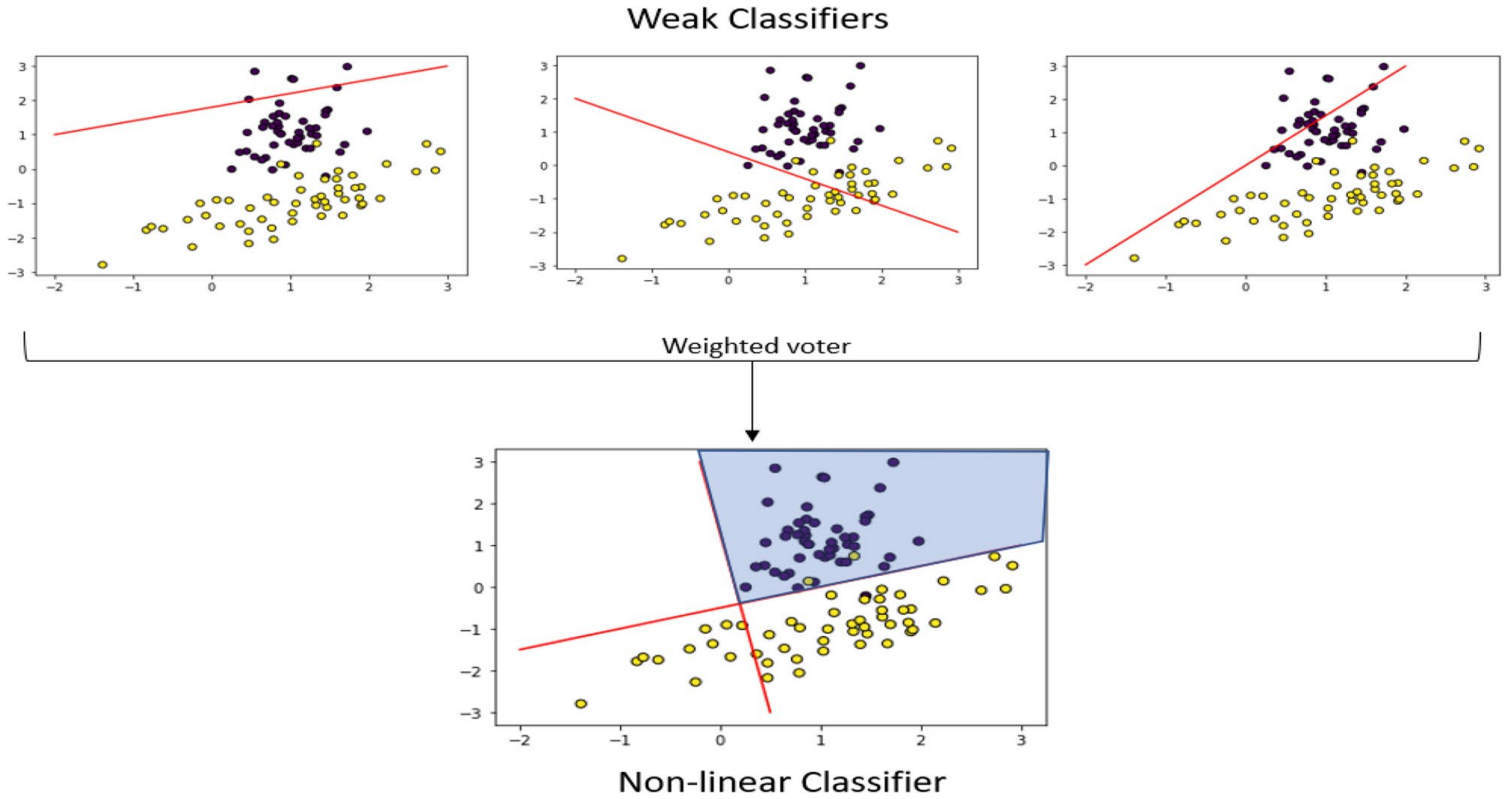
Ada Boost^38^.

An ensemble learning algorithm adjusts the weak classifier’s weights by iterating over them to enhance performance and create a more robust classifier. As shown in Fig. 5, the algorithm starts with fitting the model on the dataset and having some results, then adjusts some weights in the weak classifier and tests the model; if it is a weak classifier, it adjusts its weights till it becomes a more robust classifier.

**Figure 6:**
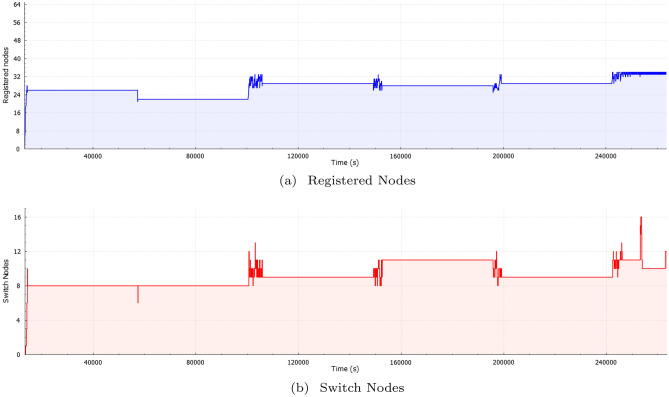
Nodes activity over time.

Figure 6 shows the number of registered nodes in a time instance such that, as part (a) and part (b) show, as the variation in the number of registered nodes increases, the variation of the number of switch nodes in a time instance increase.

### Dataset

In this work, data is collected from a test field that consists of 400 PLC modems. The PLC data are based on the PL360 chipset from Microchip, and the protocol used for the communication is PRIME standard. The data is collected using a PLC sniffer from Microchip as the sniffer is placed one node after the Data Concentrator Unit (DCU). Firstly, the data has been analyzed and filtered to be even. Then, the parameters representing the channel quality have been chosen based on the literature^5,16^.

**Figure 7:**
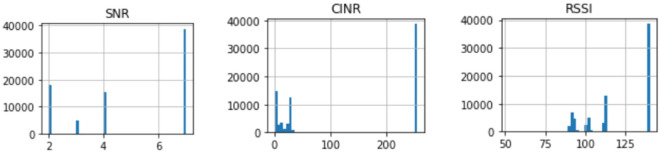
Dataset most dominant parameters histogram distribution.

**Figure 8:**
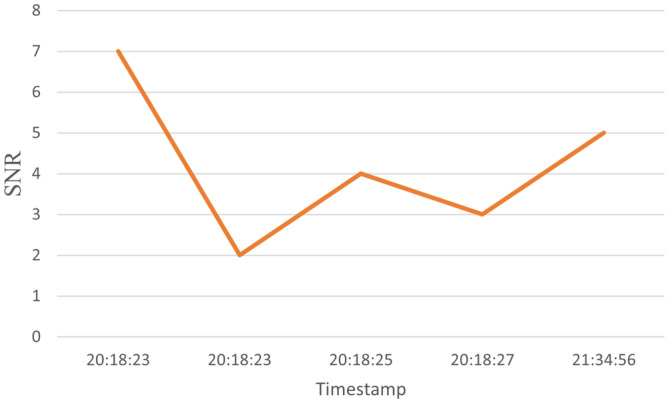
SNR values over the time.

**Figure 9:**
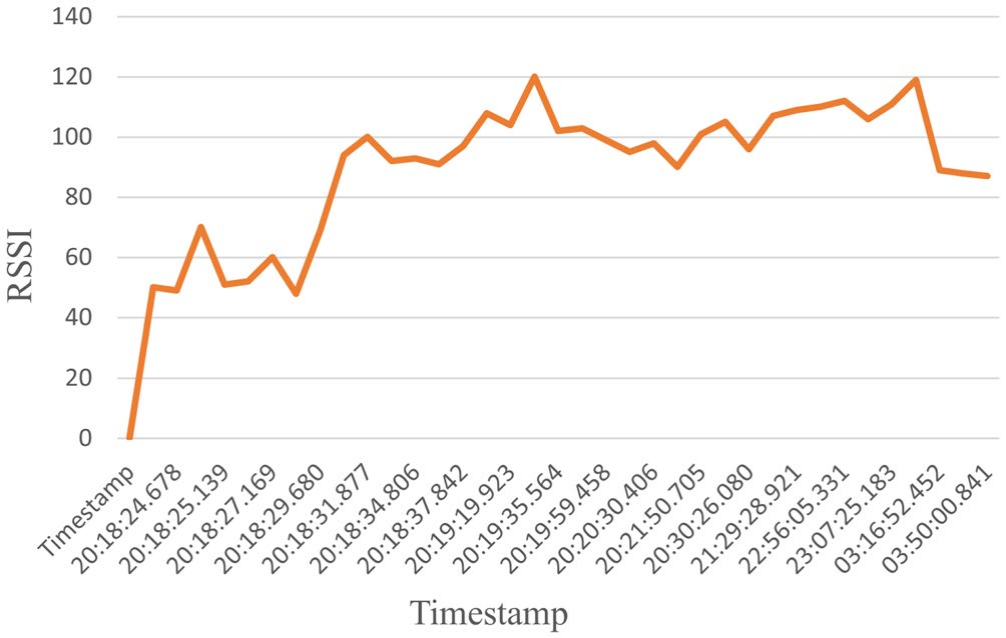
RSSI values over the time.

**Figure 10:**
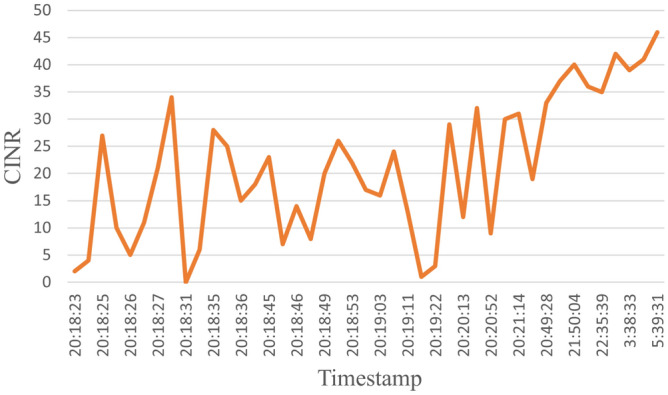
CINR values over the time.

The dataset consists of 1000 readings of the most dominant parameters, which are Signal to Noise Ratio (SNR), Received Signal Strength Indicator (RSSI), and Carrier to Interference-plus-Noise (CINR); these are the most dominant parameters as shown in Fig. 7. Table 1 shows a sample for the dataset. 50% readings for label 0 indicate that the channel is not working for these values. On the other hand, for labeled 1, the communication channel is working and suitable for data exchange. The readings helped to determine which timestamp the node was working and which was not, which helped in determining, at a given time, the probability that a node was working or not. The dataset was divided into 90% for training and 10% for testing. Samples of the data for a specific node are displayed in the following figures. For example, Fig. 8 shows the SNR values over time, while Fig. 9, depicts the RSSI of the channel for a specific node in the network. The diagrams show that the change in the parameters is random over the time of sniffing. Moreover, the CINR is illustrated in Fig. 10. The captured data shows that the signal quality is variable over time.

**Table 1:** Dataset Samples.

TimeStamp	SNR	CINR	RSSI	Label
2017-10-11 20:18:57.064	4	26	110	1
2017-10-11 20:18:25.816	2	5	50	0
2017-10-11 20:18:57.061	2	5	100	1
2017-10-11 20:18:24.678	2	4	49	0
2017-10-11 20:18:28.621	2	5	48	0
2017-10-11 20:18:56.658	3	15	103	1

### Data analysis

**Figure 11:**
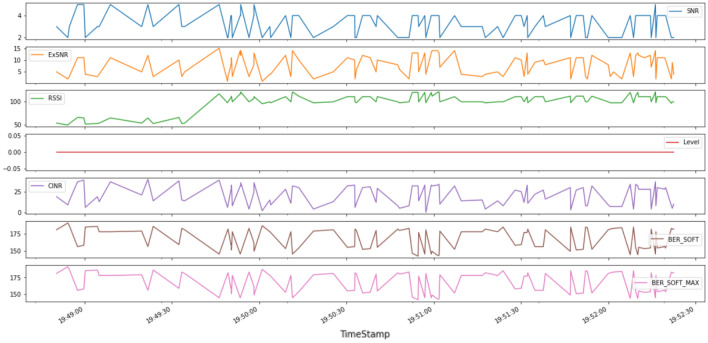
All quality features over the time.

Analysis was done on our data to identify the relation between features and how these parameters can affect nodes performance. A subset of data was taken for simplicity to visualize all feature over the time creating Figure  11. This plot clearly shows the high correlation between all features, for example when SNR increases at specific time, the Bersoft decreases so a high negative correlation appears between these two features.

**Figure 12:**
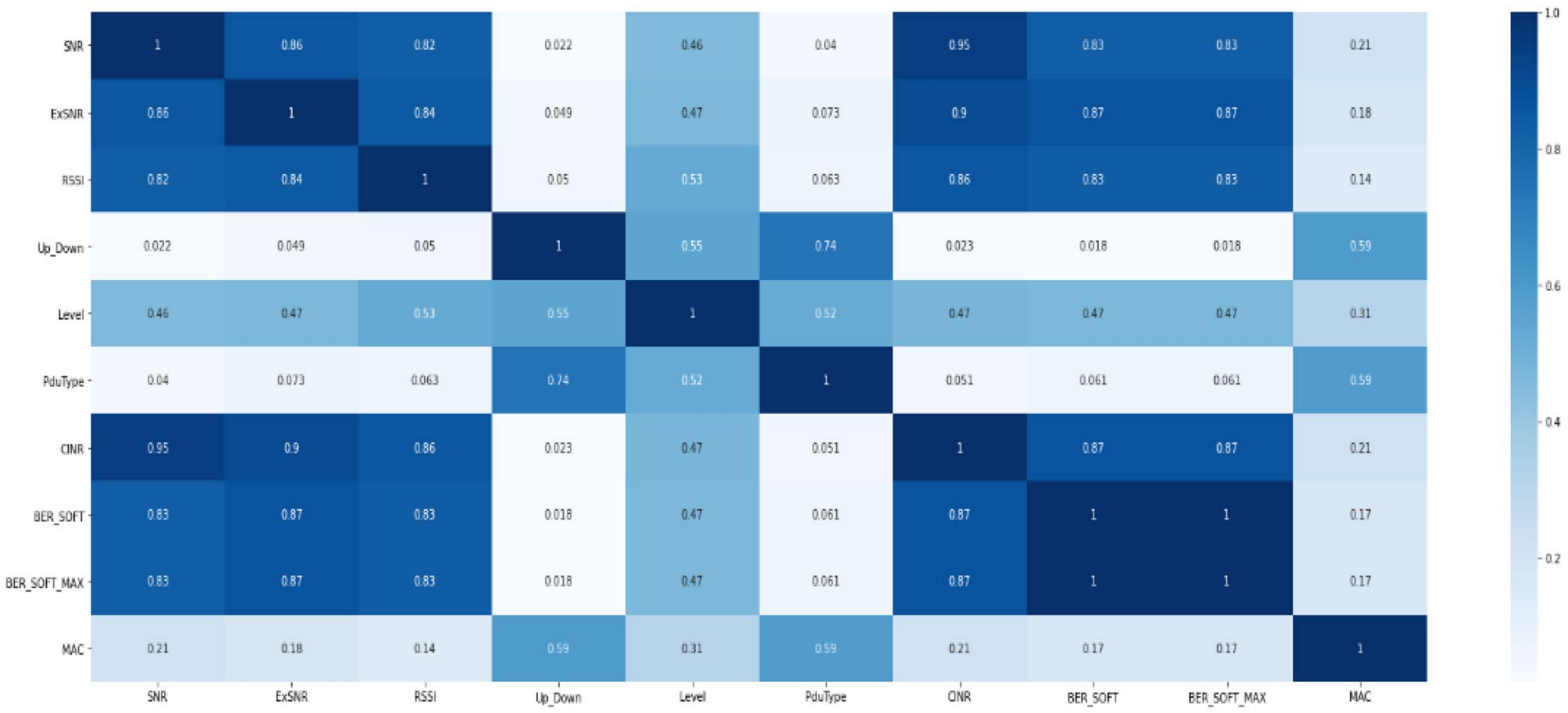
Correlation matrix of all quality features.

Another type of visualization to prove correlation is a correlation matrix which depends on Pearson correlation calculations as in Figure  12. As shown, a very high correlation appears between all quality features. Besides, a high correlation appears in up & down column with Pdutype and level. This can be explained that as a the node tends to be in a high level, lower quality appears resulting in down performance.

**Figure 13:**
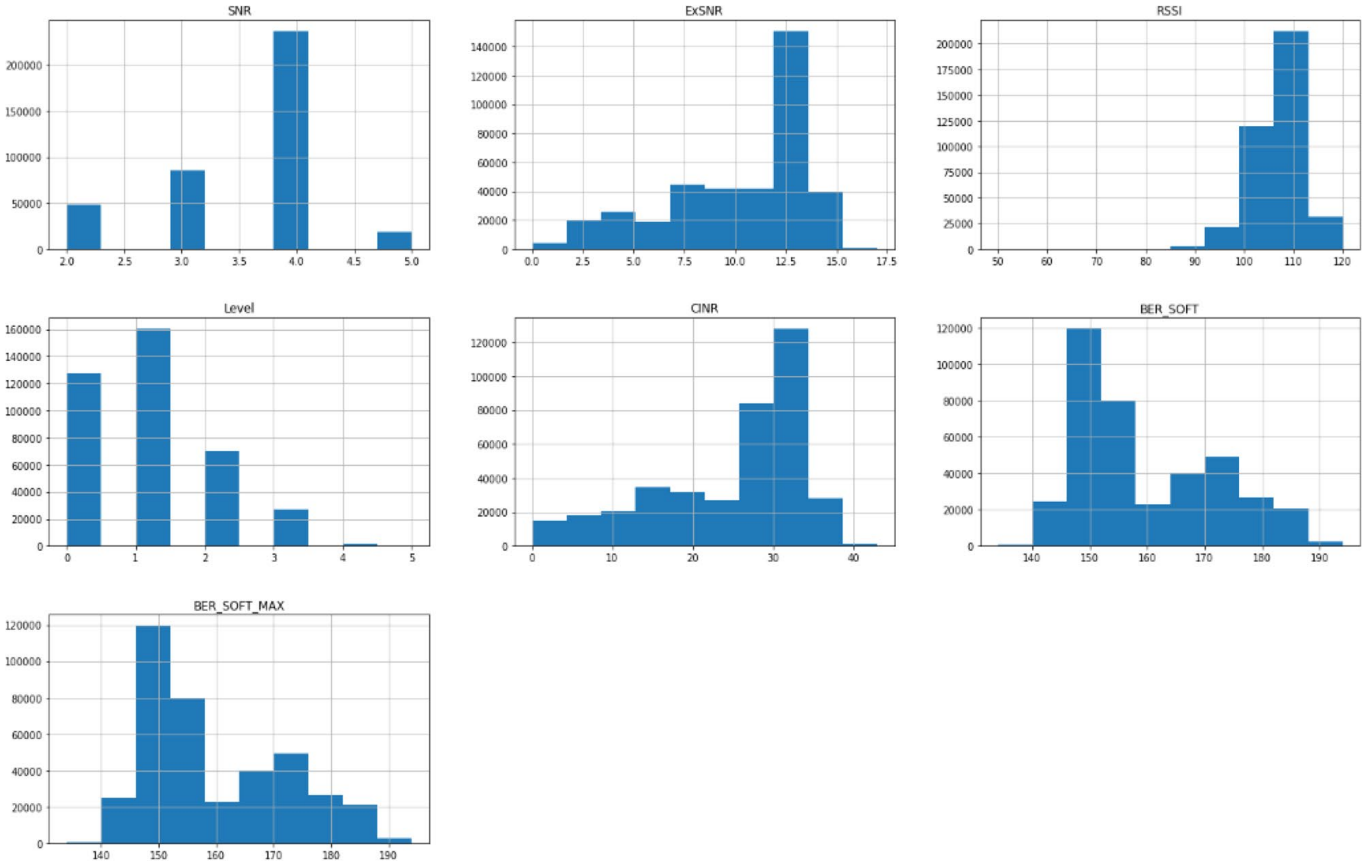
A histogram of all quality features.

A histogram of all features can be visualized as well in Figure  13. This shows that the most abundant value in SNR is 4 indicating a bad quality of almost 75% of the data. Moreover, Bersoft and Bersoft max shows the same distribution which proves their high correlation of 1 which appears in the correlation matrix.

## Results

In this section, six AI models have been used with the collected data in order to predict channel behavior. Hence, the results of these models and their impact on network performance are discussed. Then, a comparison between these results is conducted. Four metrics are used to evaluate the models; accuracy, F1-score, precision, and recall. Furthermore, a confusion matrix has been plotted for each model. The confusion matrix is a 2-d matrix whose rows refer to the true labels, and its columns refer to the predicted labels. The confusion matrix shows how many predicted instances are for each class, indicating the model performance^39^.

**Figure 14:**
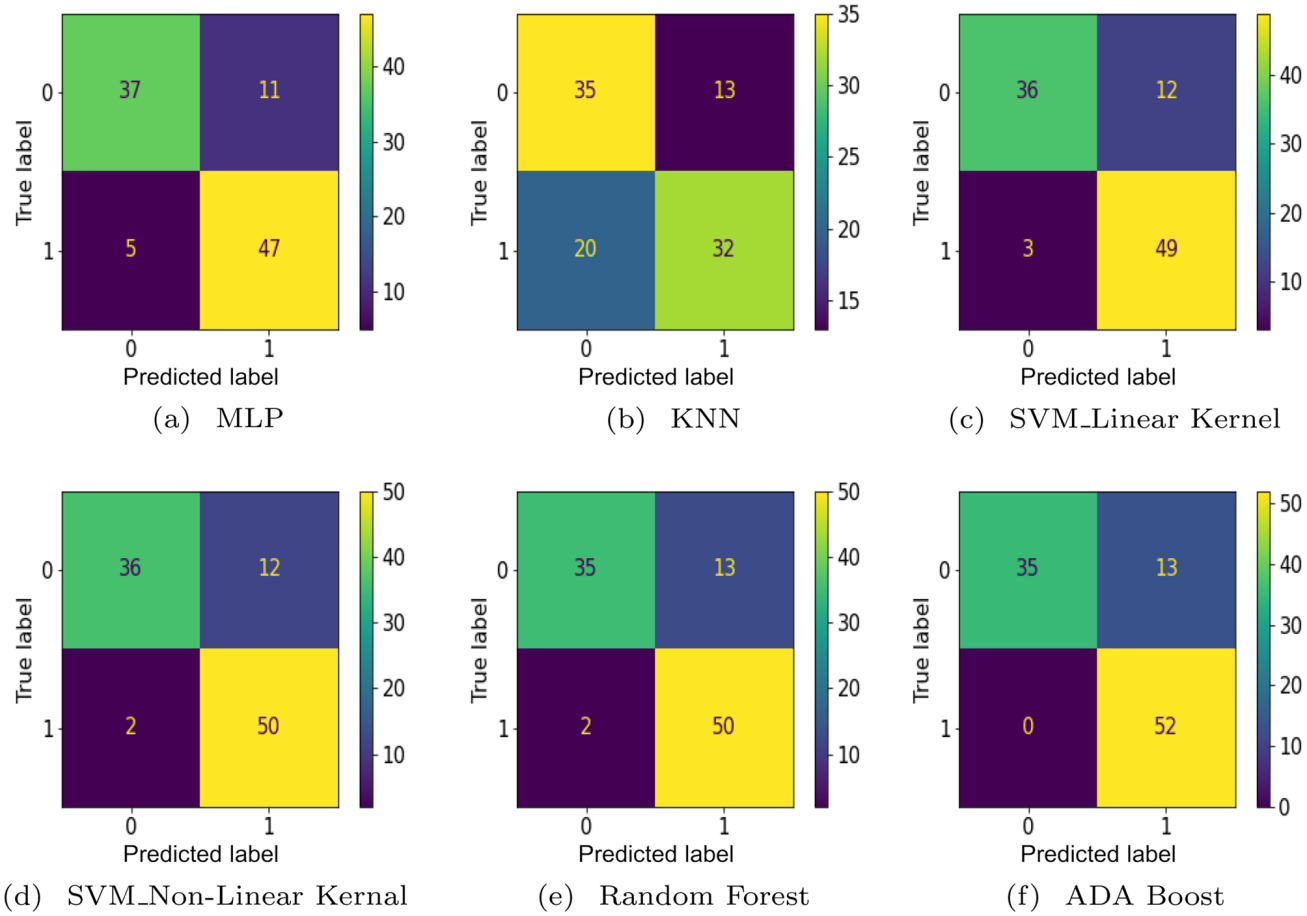
Confusion matrices for trained models.

The metrics’ equations are shown in equation 1. The accuracy is evaluated using (1a), the ratio between the summation of all the correct predictions. Which are the truly predicted positive class (TP) and truly predicted negative class (TN) to all the predictions, which are the true predictions of positive class (TP) and negative class (TN) and the falsely predicted positive class (FP) and the falsely predicted negative class (FN). As (1b) shows, the precision is how much truly predicted positive class (TP) is concerning all the predicted positive classes either truly predicted (TP) or falsely predicted (FP). The recall is how much truly predicted positive class for the number of positive class instances in the testing dataset, whether they are truly predicted (TP) or falsely predicted (FN) as shown in (1c). The F1-score is the ratio between double the multiplication of the precision and the recall to their summation as shown in (1d)^40^. Fig. 14 shows the confusion matrices for the proposed models. The confusion matrix compares the predictions of each model with respect to the actual predictions such that the rows represent the actual class and the columns represent the predicted class such that the diagonal shows the correctly classified instances.

1a $$\begin{aligned} Accuracy= & {} \dfrac{TP+TN}{TP+FP+TN+FN} \end{aligned}$$

1b $$\begin{aligned} Precision= & {} \dfrac{TP}{TP+FP} \end{aligned}$$

1c $$\begin{aligned} Recall= & {} \dfrac{TP}{TP+FN} \end{aligned}$$

1d $$\begin{aligned} F1-score= & {} 2\dfrac{precision. recall}{precision + recall} \end{aligned}$$

### Multi-layer perception (MLP)

MLP has been tested on 100 instances and achieved an accuracy of 84% such that, as Fig. 14 - a shows, 47 of 52 instances were correctly classified as class ’1’, and 37 of 48 were correctly classified as class ’0’. The model resulted in a precision of 0.8456, a recall of 0.8373, and an F1-score of 0.8384.

### K-nearest neighbour (KNN)

KNN prediction accuracy differs with different Ks, as explained in Sect. 2. Therefore, the model has been tested to predict the testing set instances with different Ks to determine the value with the best results, such that at K equals 15, the model has the best results. Fig. 14 - b shows the KNN model’s predictions when tested on 100 instances. The model correctly predicts 35 out of 48 for the 0 class and 32 out of 52 for the 1 class, which have an accuracy of 67 %, F1-score equals 0.6697, a precision of 0.6737, and recall equal of 0.6723.

### SVM-linear kernal

The model correctly predicted 49 out of 52 instances to be class ’1’ and 36 out of 48 class ’0’ as shown in Fig. 14 - c, with an accuracy of 85%, F1-score equals 0.8465, the precision equals 0.8698, and recall of 0.8454.

### SVM-non-linear kernel

The model’s accuracy is 86% which classified 50 out of 52 instances class ’1’, and 36 out of 48 of class ’0’, as shown in Fig. 14 - d, with F1-score equals 0.8572, precision of 0.8769, and recall of 0.8558

### Random forest

The Random Forest model has been tested with several estimators ranging from 1 to 100. The number of estimators is the number of trees in a random forest. This has been done to know which number of estimators will have the best results. The number of estimators that has the best accuracy is 34. Fig. 14 - e shows that the model has successfully correctly predicted 50 out of 52 instances to be class 1 and 35 out of 48 instances to be class 0. The model has an accuracy equal to 85%, F1-score of 0.8465, precision equal to 0.8698, and recall of 0.8454.

### ADA boost

The model has been tested with several estimators such that the estimators starting from 12 have the best results. Fig. 14 - f shows that the model has successfully correctly predicted 52 out of 52 instances to be class 1 and 35 out of 48 instances to be class 0. The model has an accuracy equal to 87%, F1-score of 0.8661, precision equal to 0.9, and recall of 0.8646. As shown in Table 2, The ADA Boost was the best algorithm with respect to the accuracy, F1-score, precision, and recall to predict at which time, a PLC node is optimum with an accuracy of 87%, while the least accurate model was KNN with 67% accuracy.

## Discussion

Recently, PLC has been used in different IoT applications. However, the PLC environment is vulnerable to noise sources, negatively impacting network quality. Indeed, a considerable effort has been made over the past decade to improve the network, and link quality^10,12^. For example, some researchers targeted different MAC and PHY layer implementations to improve network reliability. Furthermore, some effort has been made on the level of electronic circuit implementations^10^. However, despite the previous work on improving the network quality, PLC-based networks still need more stability due to variable conditions over time. Hence, this work uses AI to predict network stability and link quality. Six AI techniques have been used to predict the network quality and the optimum time slot for communications. Table 2 illustrates a comparison between the different techniques. The ADA Boost gave an accuracy of 87% for hitting the optimum communication slot, while KNN gave the worst accuracy of 67%. However, KNN is the fastest execution time (0.0039 sec.) for 21 threads. On the other hand, ADA Boost takes (0.02 sec) for 25 threads during the training time. This means the KNN requires fewer CPU resources than ADA Boost during the training process. This enables the concept of enabling training in a limited resources environment. Furthermore, the SVM-nonlinear kernel gave an accuracy of 86% with a training time of 0.043 sec. for 23 threads. However, SVM linear kernel achieves an accuracy of 85% for a training time of 0.024 sec. The significant advantage of selecting the optimum time slot for communication is increasing the efficiency of the communication link. This also increases the number of nodes the same DCU device can serve. Furthermore, increasing the link efficiency minimizes the number of trials to get the reading for the PLC node. Hence, the system can increase the number of nodes

**Table 2:** Results comparison.

Algorithm	Accuracy (%)	F1-Score	Precision	Recall	Training time (sec.)	Memory(MB)	Process CPU Threads	Correctly Classified Instances	
								0 out of 48	1 out of 52
MLP	84	0.8384	0.8456	0.8373	0.359787	0.523651	25 threads/31.902 sec.	37	47
KNN	67	0.6697	0.6737	0.6723	0.003982	0.539646	21 threads/7.998 sec.	35	32
SVM Linear Kernal	85	0.8465	0.8698	0.8454	0.024401	0.545628	21 threads/2.885 sec.	36	49
SVM Non-Linear Kernal	86	0.8572	0.8769	0.8558	0.043466	0.549362	23 threads/1.775 sec.	36	50
Random Forest	85	0.8465	0.8698	0.8454	0.086674	0.551956	21 threads/2.292 sec.	35	50
ADA Boost	87	0.8661	0.9	0.8646	0.020187	0.553989	25 threads/74.03 sec.	35	52

served by the same DCU.

## Conclusion

Predicting the availability of a PLC node earlier enhances the network performance. MLP, KNN, SVM linear and non-linear kernels, Random Forest, and AdaBoost algorithms were trained and tested to predict whether a PLC node was available at a particular time or not. They represent Statistical, Vector-based, regression, decision, and predictive algorithms. Signal to Noise Ratio (SNR), Received Signal Strength Indicator (RSSI), and Carrier to Interference-plus-Noise (CINR) readings were used to determine whether a PLC node was optimum to be used at a specific time using a dataset of 1000 instances was used such that 90% of it was used in training the model. The model has achieved accuracy, F1-score, precision, and recall which are 87%, 0.86613, 0.9, and 0.8646, respectively, for the AdaBoost algorithm, which exceeded the other algorithms.

## Data Availability

The datasets generated during and/or analyzed during the current study are available from the corresponding author upon reasonable request.
